# Mortality of neurological disorders in Tanzania: analysis of baseline data from sample vital registration with verbal autopsy (SAVVY)

**DOI:** 10.1080/16549716.2019.1596378

**Published:** 2019-05-30

**Authors:** Francis Levira, Charles R. Newton, Honorati Masanja, Peter Odermatt

**Affiliations:** a Department of Epidemiology and Public Health, Swiss Tropical and Public Health Institute, Basel, Switzerland; b University of Basel, Basel, Switzerland; c Health Systems, Impact Evaluation, and Policy, Ifakara Health Institute, Dar-es-Salaam, Tanzania; d Kenya Medical Research Programme-Wellcome Trust Collaborative Programme, Kilifi, Kenya; e Department of Psychiatry, University of Oxford, Oxford, UK

**Keywords:** Cerebrovascular, epilepsy, meningitis, cerebral palsy, intrauterine hypoxia

## Abstract

**Background**: Neurological disorders (ND) have a profound consequence on human productivity, quality of life and survival. There are limited data on the burden of ND in Tanzania due to insufficient coverage of civil and vital registration systems.

**Objectives**: This study was conducted to estimate mortality of ND in all ages in Tanzania using data from the Sample Vital Registration with Verbal Autopsy (SAVVY) study.

**Methods**: Multistage random sampling was employed to select 23 districts, 1397 census enumeration areas and 154,603 households. During the baseline survey conducted between 2011 and 2014, deaths which occurred 12 months prior to the baseline survey were documented followed by verbal autopsy interviews. Causes of death were certified using International Classification of Diseases.

**Results**: The baseline survey enrolled a total of 650,864 residents. A total of 6645 deaths were reported to have occurred 12 months before the date of survey. Death certification was available for 5225 (79%) deaths. The leading causes of death were cerebrovascular diseases with a cause-specific mortality fraction (CSMF) of 1.64% (95% CI: 1.30–1.99) and 3.82% (95% CI: 2.92–4.72) in all ages and adults older than 50 years, respectively. Stroke accounted for 92% of all cerebrovascular deaths. Mortality of epilepsy was estimated with a CSMF of 0.94% (95% CI: 0.68–1.20); meningitis with a CSMF of 0.80% (95% CI: 0.56–1.04); cerebral palsy and other paralytic syndromes with a CSMF of 0.46% (95% CI: 0.27–0.65); and intrauterine hypoxia in neonates with a CSMF of 2.06% (95% CI: 1.12–3.01). Overall, mortality of ND was estimated with a CSMF of 4.99% (95% CI: 4.40–5.58).

**Conclusions**: The SAVVY survey provides estimates of mortality burden of ND in Tanzania. The study provides a basis for monitoring trends of ND and contributes to advancing knowledge of the burden of diseases. Integrating morbidities measures into the SAVVY design will provide comprehensive measures of burden of ND taking into account lifetime disabilities created by ND.

## Background

Neurological disorders (ND) have a profound consequence on human productivity, quality of life and survival in developed and developing countries. The most prevalent ND globally include dementia (progressive memory loss); Parkinson’s (impaired motor system); multiple sclerosis (problem with vision, movement, sensation or balance); epilepsy (sudden recurrence of unprovoked seizures); and stroke (two or one sides paralysis or numbness).

Dementia and Parkinson’s disease are the most common ND in most developed countries with a reported increasing trend [1]. The increases in ND’s mortality in developed countries are higher than increases in other chronic diseases such as cancer and diseases of circulation [2]. Substantial proportions of neurological disorders in developed countries are thought to be attributed to increased life expectancy (aging) and unhealthy lifestyle [3].

In most developing countries, on the other hand, etiological studies reported that most ND originate from infections of the central nervous system (CNS) and brain trauma [4–7]. The most common reported ND in sub-Saharan Africa (SSA) include stroke, epilepsy, meningitis, paraparesis, neuropathies, and traumatic brain injuries [8–10].

In 2010, Global Burden of Disease (GBD) report indicated that non-communicable diseases (NCD) accounted for 54% of global burden of disease (morbidities and premature mortality); an increase from 43% in 1990 [11]. With the rapid epidemiological transition in developing countries, mortalities associated with age-related and lifestyle ND are expected to increase in the presence of infection-related ND; therefore, deliberate efforts in understanding the epidemiology of ND is necessary.

The morbidity and mortality burden of stroke as an example is reported to increase in developing countries, accounting for 75% of global deaths and 81% stroke-related Disability Adjusted Life Years (DALYs) [12].

In SSA, there is a paucity of national data on the epidemiology of ND. Most references of national estimates of the burden of NDs are cited from the modeled estimates of GBD study. However, for most developing countries, input parameters used in modeling are obtained from inadequate vital registrations records or small local studies. Coverage of mortality and morbidity for China and India as an illustration was only 15% and 1%, respectively, for mental, neurological and substance abuse at the time GBD for the year 2013 was estimated [13]. Model-based estimates from GBD not only are free from coverage limitations but also considerable reliance on geospatial data and experts' opinions [12]. Global estimates in some countries and cases deviate from the true epidemiological profile and thus are deemed unreliable for local planning by policy makers [14].

Tanzania lacks national mortality data on ND due to the insufficient coverage of civil and vital registration systems as in many SSA countries [15,16]. Poor coverage of civil and vital registration systems at a national level and over-reliance on global estimation have concealed crucial statistics necessary in understanding population health, improving health outcomes and monitoring disease trends [17,18]. Several studies have linked higher coverage of civil and vital statistics with improved health outcomes in several countries [18].

In an effort to increase utilization of local data for comprehensive health planning, the Tanzanian government was supported by development partners to develop and implement a SAmple Vital registration with Verbal AutopsY (SAVVY) study aimed at providing nationally representative estimates of mortality and causes of death by sex, residence, and zone [19]. Nearly 75% of deaths occur at home in most developing countries and cause of death determination is nearly absent in Tanzania as it is in many low- and middle-income countries. Alternatively, the verbal autopsy (VA) process facilitates generation of causes of death statistics in situations where post-mortem examinations and death certification are not routinely conducted [20–22]. VA remained the only tool reliable for providing vital statistics information.

We examined the SAVVY data to estimate mortality rates of the neurological disorders by sex, age, residence, and zones in Tanzania.

## Material and methods

### Design and sampling

**Figure 1.**
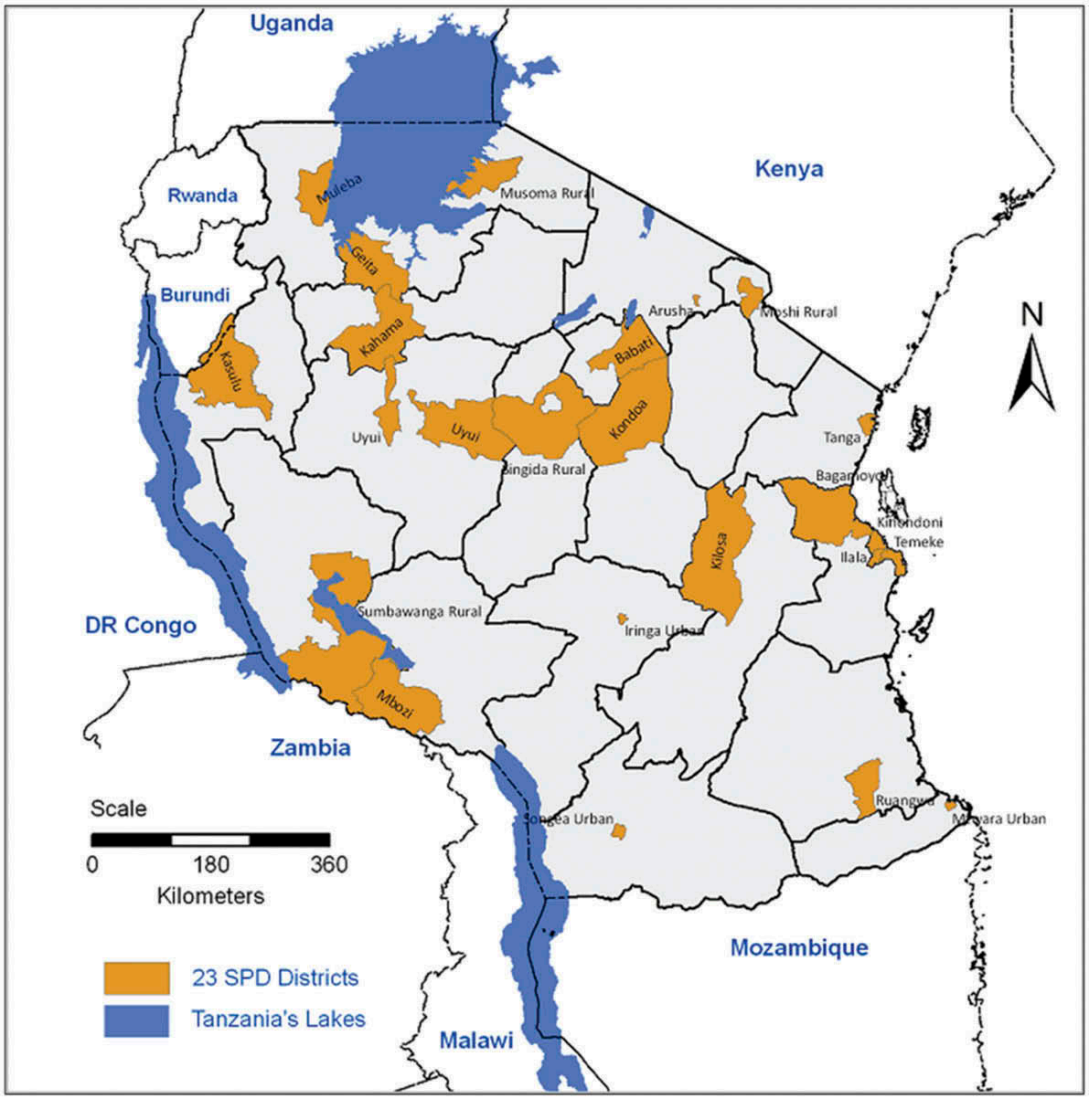
Geographic locations of 23 SAVVY districts on a map of Tanzania.

SAVVY is a community-based system implemented in a nationally representative cluster sample [19]. Multistage random sampling was employed to select 23 districts, 1397 enumeration areas, and 154,603 households from mainland Tanzania, stratified by residency and zones to meet the proposed overall sample. The sampling frame for this study was based on the 2002 Population and Housing Census for Tanzania Mainland. A full description of design and methods has been published elsewhere [23]. Figure 1 shows the map of Tanzania with shaded sampled districts.

### Baseline census

Baseline censuses were sequentially conducted in 23 districts between 2011 and 2014 in order to establish denominators for estimating different demographic indicators. During the baseline census, the sociodemographic information of the household members was collected which included: age, sex, education, and relationship to head of household. Deaths that had occurred during the 12 months prior to the census date were retrospectively documented in all households.

### Verbal autopsy

Verbal autopsy (VA) interviews were conducted for all documented deaths by trained SAVVY VA coordinators with the most appropriate person in the household after setting up an appointment. Standard verbal autopsy questionnaires (2007 version) that have been developed by the World Health Organization (WHO) in collaboration with other stakeholders were used [24]. Completed VA questionnaires were sent to physicians for a cause of death certification.

### Physician’s assignment of causes of deaths

The cause of death for each interviewed death was determined using the International Classification of Diseases, tenth revision (ICD-10), as published by the WHO [25]. Each VA questionnaire of the deceased was reviewed independently by two physicians to ascertain causes of deaths and produce death certificates. In case of discrepancies, two independent physicians met to resolve the discrepancies. If disagreed, the cause of death was declared undetermined (meaning there was no sufficient information to determine the cause of death). Physicians reviewing VA questionnaires were medical doctors (not neurologists) independent of the research institution trained on death certification using ICD-10 classification.

Death certificates were requested and documented during VA interviews. However, these certificates were rarely available and coded in ICD-10; therefore, for consistency reasons, we did not consider these certificates. Neurological causes of deaths were classified using code ranges of G00–G99 for diseases of nervous system, I60–69 for cerebrovascular diseases, P20 for intrauterine hypoxia, C71 for malignant neoplasm of brain, D33 for benign neoplasm of brain and other parts of the CNS, Q00–Q07 for congenital malformations of the nervous system, S06 for traumatic brain injuries/intracranial injury, and R25–R29 for symptoms and signs involving the nervous and musculoskeletal systems [26,27].

### Statistical analysis

National Population and Housing Census data were used to provide a basis for data weighting. The gross weight was estimated as the product of reciprocal of the probability of selection of districts within zones, enumeration area (EA) within districts and households within EA. Mortality rate was estimated as a weighted ratio of the number of deaths and population. Cause-specific mortality fraction (CSMF) was calculated as a ratio of deaths due to a specific cause over the total number of deaths for which cause of death information was available. CSMF were compared across the subpopulation by calculating the mortality rate fraction (MRF), which is the ratio of two CSMF. Females, urban, and age 50 and above and lake zone were set as the reference category for CSMF comparisons. Lake zone is known to have poor intervention coverage for a substantial number of health systems performance indicators; therefore, we set it as a reference category [28].

## Results

**Figure 2.**
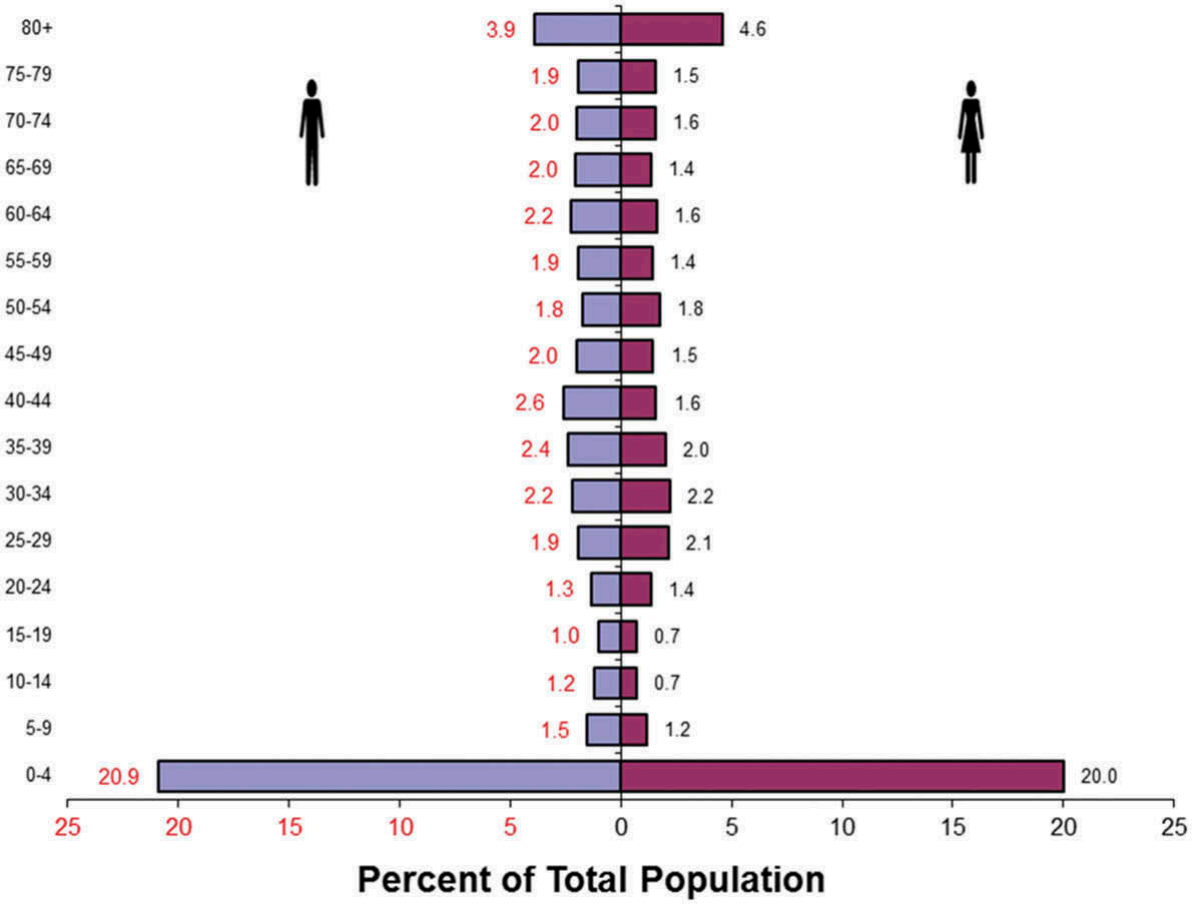
Age distribution of 6645 reported deaths (3509 males and 3136 females) from SAVVY districts.

A total of 650,864 residents in 154,603 households were enrolled during the baseline census survey. There were 91,329 (51%) households in rural areas and 63,274 (41%) in urban areas. The majority of households were headed by males (72%). The average household size was 4.5 in rural areas and 3.7 in urban areas. A total of 6645 deaths were documented to have occurred 12 months prior to the baseline survey corresponding to an annual weighted crude death rate of 10.8 (95% CI: 10.8–10.9) deaths per 1000 population. Teh age distribution of the reported deaths is shown in death pyramid in Figure 2.

VA interviews were conducted for 6608 (99%) of the documented 6645 deaths. Causes of death certification were completed for 5225 (79%) of 6608 VA interviewed deaths. Of 5225 deaths with complete cause of death, 872 (17%) were newborn aged 0–29 days (VA form 1), 1096 (21%) were children aged 29 days−14 years (VA form 2) and 3257 (62%) were adults older than 15 years (VA form 3). Causes of death could not be ascertained for 1383 deaths due to absence of a reliable caregiver to respond to the VA interview, incomplete VA interviews, and incomplete cause of death determination by physicians, among other things.

### Causes of neurological deaths

A total of 261 neurological deaths were determined; 72 (27.6%) were children younger than 5 years, 22 (8.4%) were children aged 5–19 years, 65 (24.9%) were adults aged 20–49 years, and 102 (39.1%) were adults older than 50 years. All neurological deaths were coded into 30 three- or four-digit ICD-10 causes and later grouped into nine major categories of cerebrovascular diseases, meningitis, epilepsy, cerebral palsy and other paralytic syndromes, intrauterine hypoxia, malignant and benign neoplasms of brain and CNS, congenital malformations of the nervous system, other neurological disorders, and symptoms and signs involving the nervous and musculoskeletal systems (Table 1).

**Table 1.** Distribution of causes of neurological death by age group.

	Age group				
	<5	5–19	20–49	50+	All ages
**Cerebrovascular diseases**	**1.4**		**27.7**	**65.7**	**33.0**
Stroke, not specified as hemorrhage or infarction	1.4		24.6	60.8	30.3
Hypertensive encephalopathy				1.0	0.4
Sequelae of stroke, not specified as hemorrhage or infarction			3.1	2.9	1.9
Sequelae of other and unspecified cerebrovascular diseases				1.0	0.4
**Epilepsy**	**4.2**	**40.9**	**36.9**	**11.8**	**18.4**
Other generalized epilepsy and epileptic syndromes				1.0	0.4
Epilepsy, unspecified	4.2	40.9	36.9	11.8	18.4
**Meningitis**	**34.7**	**22.7**	**10.8**	**4.9**	**16.1**
Bacterial meningitis, not elsewhere classified	4.2				1.2
Meningitis due to other and unspecified causes	1.4				0.4
Meningitis, unspecified	29.2	22.7	10.8	4.9	14.6
**Cerebral palsy and other paralytic syndromes**	**11.1**	**18.2**	**3.1**	**9.8**	**9.2**
Cerebral palsy	1.4				0.4
Cerebral palsy, unspecified	8.3	13.6			3.5
Hemiplegia, unspecified				4.9	1.9
Paraplegia, unspecified		4.6	1.5	2.9	1.9
Paralytic syndrome, unspecified	1.4		1.5	2.0	1.5
**Intrauterine hypoxia**	**25.0**				**6.9**
**Malignant and benign neoplasms of brain and CNS**		**9.1**	**7.7**	**1.0**	**3.1**
Malignant neoplasm of brain			3.1		0.8
Malignant neoplasm of spinal cord, cranial nerves, and other parts of CNS		9.1	3.1	1.0	1.9
Benign neoplasm of brain and other parts of central nervous system			1.5		0.4
**Congenital malformations of the nervous system**	**12.5**		**1.5**		**3.8**
Congenital hydrocephalus	1.4				0.4
Congenital hydrocephalus, unspecified	4.2		1.5		1.5
Spina bifida	1.4				0.4
Spina bifida, unspecified	4.2				1.2
Congenital malformation of nervous system, unspecified	1.4				0.4
**Other neurological disorders**	**4.2**		**6.2**	**2.9**	**3.8**
Migraine				1.0	0.4
Migraine, unspecified			3.1	1.0	1.2
Polyneuropathy, unspecified (neuropathy NOS)			1.5	1.0	0.8
Hydrocephalus	4.2		1.5		1.5
**Symptoms and signs involving the nervous** **and musculoskeletal systems**	**6.9**	**9.1**	**6.2**	**2.9**	**5.4**
Other and unspecified symptoms and signs involving the nervous and musculoskeletal systems				1.0	0.4
Headache			6.2	2.0	2.3
Other and unspecified convulsions	6.9	9.1			2.7
**Number of deaths**	**72**	**22**	**65**	**102**	**261**

### Neurological disorders mortality

Of the reported ND-related deaths, the leading causes were cerebrovascular diseases (33.0%), epilepsy (18.4%), meningitis (16.1%), cerebral palsy and other paralytic syndromes (CP) (9.2%), and intrauterine hypoxia (IH) (6.9%). All causes mortality of ND was estimated with a CSMF of 4.99% (95% CI: 4.40–5.59) (Table 2). There were no differences in CSMF among males (4.76%) and females (5.27%) (MRF = 0.90, 95% CI: 0.70–1.16). Compared to adults aged 50 years and above, mortality of ND was estimated to be lower among children aged 0–4 (MRF = 0.71, 95% CI: 0.52–0.97), and comparable in children aged 5–19 (MRF = 1.07, 95% CI: 0.64–1.72) and adults aged 20–49 (MRF = 1.07, 95% CI: 0.64–1.72). Mortality was lower by 23.0% in urban (4.35%) than rural (5.63%) areas (MRF = 0.77, 95% CI: 0.40–0.99). Mortality of ND was comparable across zones. Using census population and crude death rate of 2012 and CSMF observed in this study, we estimated the number of neurological deaths to range from 18,000 to 22,000 in Tanzania in 2012.

**Table 2.** Cause-specific mortality fraction (%) estimates of neurological disorders and epilepsy by sex, age, residency, and zone.

	Neurological disorders			Epilepsy		
	Deaths	CSMF (95% CI)	MRF (95% CI)	Deaths	CSMF (95% CI)	MRF (95% CI)
**Sex**						
Males	134	4.76 (4–5.55)	0.90 (0.70–1.16)	36	1.28 (0.86–1.69)	2.37 (1.23–4.88)
Females	127	5.27 (4.38–6.16)		13	0.54 (0.25–0.83)	
**Age**						
<5	72	4.13 (3.19–5.06)	0.71 (0.52–0.97)	3	0.17 (0–0.37)	0.23 (0.04–0.84)
5–19	22	6.25 (3.72–8.78)	1.07 (0.64–1.71)	9	2.56 (0.91–4.20)	3.45 (1.30–8.72)
20–49	65	4.72 (3.6–5.84)	0.81 (0.58–1.12)	24	1.74 (1.05–2.43)	2.35 (1.15–5.03)
50+	102	5.82 (4.72–6.91)		13	0.74 (0.34–1.14)	
**Residency**						
Rural	149	5.63 (4.75–6.50)		30	1.13 (0.73–1.54)	0.65 (0.35–1.19)
Urban	112	4.35 (3.56–5.13)	0.77 (0.60–0.99)	19	0.74 (0.41–1.07)	
**Zone**						
Western	31	4.74 (3.11–6.37)	0.75 (0.46–1.21)	7	1.07 (0.28–1.86)	1.00 (0.31–3.16)
Northern	25	3.53 (2.17–4.88)	0.56 (0.33–0.93)	3	0.42 (0–0.90)	0.40 (0.07–1.65)
Central	43	4.68 (3.31–6.04)	0.74 (0.48–1.15)	12	1.30 (0.57–2.04)	1.22 (0.46–3.45)
Southern Highlands	39	6.61 (4.60–8.61)	1.05 (0.67–1.64)	4	0.68 (0.01–1.34)	0.63 (0.14–2.37)
Eastern	43	4.8 (3.41–6.21)	0.77 (0.49–1.18)	8	0.89 (0.28–1.51)	0.84 (0.27–2.56)
Southern	33	4.65 (3.10–6.20)	0.74 (0.46–1.18)	7	0.99 (0.26–1.71)	0.92 (0.28–2.91)
Lake	47	6.27 (4.54–8.01)		8	1.07 (0.33–1.80)	
**Overall**	**261**	4.99 (4.40–5.58)		**49**	0.94 (0.68–1.20)	

Ref: Reference category; CSMF: cause-specific mortality fraction, MRF, mortality rate fraction; CI: confidence interval.

### Cerebrovascular mortality

Cerebrovascular diseases are a category of a broader group of diseases of the circulatory system. Cerebrovascular diseases accounted for 19.0% of all deaths from diseases of circulation, after hypertension which accounted for 66.0% of deaths from diseases of circulation. A total of 86 cerebrovascular deaths were reported out of 5225 certified deaths. Among those who died of cerebrovascular diseases, stroke was the leading cause of death, constituting 92.0% of all reported cerebrovascular deaths. The remaining 8.0% constituted hypertensive encephalopathy, sequelae of stroke not specified as hemorrhage or infarction, and sequelae of other unspecified cerebrovascular diseases.

The mortality of cerebrovascular-related deaths was estimated with a CSMF of 1.64% (95% CI: 1.30–1.99); with a higher estimate in adults older than 50 years (3.82%) than adults aged 20–49 (1.31%) (MRF = 3.4, 95% CI: 1.19–5.58) (Table 2). There was a comparable estimate in urban (1.98%) and rural (1.32%) populations (MRF = 1.5, 95% CI: 0.95–2.37). No significant differences were observed between lake zone and other zones.

In adults older than 50 years, the mortality of cerebrovascular-related deaths was estimated with a CSMF of 3.82% (95% CI: 2.92–4.72). CSMF was lower in males (2.87%) than females (4.93%) (MRF = 0.58, 95% CI: 0.34–0.97), comparable in urban (4.19%) and rural (3.42%) (MRF = 1.23, 95% CI: 0.73–2.06), and lower in central zone (1.68%) compared to lake zone (6.22%) (MRF = 0.27, 95% CI: 0.08–0.76).

### Epilepsy mortality

Of all ND-related deaths, epilepsy accounted for 18.4%; 4.2% among children < 5 years, 40.9% among children aged 5–19, 36.9% among adults aged 20–49, and 11.8% among adults older than 50 years (Table 1). Unspecified convulsive epilepsy (98.0%) and other generalized epilepsy and unspecified epileptic syndromes (2.0%) were the category reported for all deaths.

Of 5225 certified deaths, 49 were epilepsy-related with a CSMF of epilepsy of 0.94% (95% CI: 0.68–1.20) with significantly higher estimates in males (1.28%) than females (0.54%) (MRF = 2.37, 95% CI: 1.23–4.88) (Table 3). Epilepsy mortality was higher in children aged 5–19 (MRF = 3.45, 95% CI: 1.30–8.72) and adults aged 20–49 (MRF = 2.35, 95% CI: 1.15–5.03) than adults aged 50 years and above. Epilepsy mortality was observed to be comparable in urban (0.74%) and rural (1.13%) areas (MRF = 0.65, 95% CI: 0.35–1.19). Epilepsy mortality was also comparable across zones.

**Table 3.** Cause-specific mortality fraction (%) estimates of cerebrovascular disorders (95% CI) by sex, age, residency, and zone.

	Cerebrovascular diseases			Cerebrovascular diseases (50+ years)		
	Deaths	CSMF (95% CI)	MRF (95% CI)	Deaths	CSMF (95% CI)	MRF (95% CI)
**Sex**						
Males	38	1.99 (1.43–2.55)		27	2.87 (1.80–3.93)	0.58 (0.34–0.97)
Females	48	1.35 (0.92–1.78)	0.68 (0.43–1.06)	40	4.93 (3.44–6.42)	
**Age**						
<5	1	0.06 (0.00–0.17)	0.02 (0.00–0.09)			
5–19	0					
20–49	18	1.31 (0.71–1.91)	0.34 (0.19–0.58)			
50+	67	3.82 (2.92–4.72)				
**Residency**						
Rural	35	1.32 (0.89–1.76)		29	3.42 (2.20–4.65)	
Urban	51	1.98 (1.44–2.52)	1.50 (0.95–2.37)	38	4.19 (2.89–5.50)	1.23 (0.73–2.06)
**Zone**						
Western	11	2.14 (1.10–3.17)	0.79 (0.33–1.81)	9	6.08 (2.23–9.93)	0.99 (0.37–2.47)
Northern	9	1.27 (0.44–2.09)	0.59 (0.23–1.43)	7	2.30 (0.62–3.99)	0.37 (0.12–1.00)
Central	8	0.87 (0.27–1.47)	0.41 (0.15–1.01)	6	1.68 (0.35–3.01)	0.27 (0.08–0.76)
Southern Highlands	12	2.03 (0.89–3.17)	0.95 (0.41–2.14)	10	6.54 (2.62–10.45)	1.05 (0.41–2.59)
Eastern	18	2.01 (1.09–2.93)	0.94 (0.45–1.98)	12	3.87 (1.72–6.02)	0.62 (0.26–1.48)
Southern	12	1.69 (0.74–2.64)	0.79 (0.34–1.78)	10	3.68 (1.44–5.91)	0.59 (0.23–1.46)
Lake	16	1.68 (0.70–2.67)		13	6.22 (2.94–9.49)	
**Overall**	**86**	**1.64 (1.30–1.99)**		**67**	**3.82 (2.92–4.72)**	

Ref: Reference category; CSMF: cause-specific mortality fraction; MRF: mortality rate fraction; CI: confidence interval.

### Meningitis mortality

Bacterial meningitis accounted for 16.1% of all ND deaths; 34.7% among children < 5 years, 22.7% among children aged 5–19, 10.8% among adults aged 20–49, and 4.9% among adults older than 50 years (Table 1). No viral-related meningitis deaths were identified by physicians.

Of certified deaths, meningitis-related mortality was estimated with a CSMF of 0.80% (95% CI: 0.56–1.04) (Table 4). Compared to adults older than 50 years (CSMF = 0.28%), meningitis mortality was high in children 0–4 (1.43%) (MRF = 5.02, 95% CI: 1.89–16.81) and those aged 5–19 years (1.42%) (CSMF = 4.98, 95% CI: 1.15–21.64). Meningitis mortality was comparable in urban (0.66%) and rural (0.94%) (MRF = 0.70, 95% CI: 0.35–1.35) areas and among males (1.04%) and females (0.79%) (MRF = 1.04, 95% CI: 0.54–2.04).

**Table 4.** Cause-specific mortality fraction (%) estimates of meningitis disorders by sex, age, residency, and zone.

		Meningitis			Meningitis (age 0–19)	
	Deaths	CSMF(95% CI)	MRF (95% CI)	Deaths	CSMF (95% CI)	MRF (95% CI)
**Sex**						
Males	23	0.82 (0.48–1.15)	1.04 (0.54–2.04)	18	1.57 (0.85–2.29)	1.25 (0.57–2.84)
Females	19	0.79 (0.43–1.14)		12	1.26 (0.55–1.97)	
**Age**						
<5	25	1.43 (0.88–1.99)	5.02 (1.89–16.81)		1.43 (0.88–1.99)	1.01 (0.38–3.37)
5–19	5	1.42 (0.18–2.66)	4.98 (1.15–21.64)	25	1.42 (0.18–2.66)	
20–49	7	0.51 (0.13–0.88)	1.78 (0.49–7.13)	5		
50+	5	0.28 (0.03–0.53)				
**Residency**						
Rural	25	0.94 (0.57–1.31)		19	1.57 (0.87–2.27)	
Urban	17	0.66 (0.35–0.97)	0.70 (0.35–1.35)	11	1.24 (0.51–1.97)	0.79 (0.34–1.75)
**Zone**						
Western	5	0.76 (0.10–1.43)	0.67 (0.17–2.11)	5	1.26 (0.16–2.37)	0.58 (0.15–2.01)
Northern	4	0.56 (0.01–1.11)	0.47 (0.10–1.68)	1	0.56 (0.00–1.65)	0.25 (0.01–1.91)
Central	1	0.11 (0.00–0.32)	0.09 (0.00–0.65)	0		
Southern Highlands	14	2.37 (1.14–3.60)	1.97 (0.80–5.17)	12	4.33 (1.93–6.73)	0.04 (0.02–0.03)
Eastern	5	0.56 (0.07–1.05)	0.46 (0.12–1.55)	1	0.31 (0.00–0.91)	0.14 (0.00–1.05)
Southern	4	0.56 (0.01–1.11)	0.47 (0.10–1.68)	3	1.39 (0.00–2.95)	0.64 (0.11–2.65)
Lake	9	1.20 (0.42–1.98)		8	2.18 (0.69–3.68)	
**Overall**	**42**	**0.80 (0.56–1.04)**		**30**	**1.43 (0.92–1.94)**	

Ref: Reference category; CSMF: cause-specific mortality fraction; MRF: mortality rate fraction; CI: confidence interval.

Subanalysis of meningitis mortality in children similar to the above indicated no differences in CSMF related to sex, age, residency, and zone.

### Cerebral palsy and other paralytic syndromes

CP accounted for 9.2% of all ND deaths; 11.1% among children < 5 years, 18.2% among children aged 5–19, 3.1% among adults aged 20–49, and 9.8% among adults older than 50 years (Table 1). Cerebral palsy deaths were reported in children younger than 20 years while paralytic syndromes (hemiplegia, paraplegia, and paralysis) were common in adults older than 20 years. A total of 24 deaths from CP and other paralytic syndromes were identified with a CSMF of 0.46% (95% CI: 0.27–0.65). Mortality of cerebral palsy in children younger than 5 years was estimated with a CSMF of 0.40% (95% CI: 0.10–0.70).

### Intrauterine hypoxia

A total of 18 deaths from IH were coded. IH accounted for 6.9% of all ND deaths and 25.0% among children younger than 5 years died of ND. Given the fact that IH is diagnosed at birth or the early days of neonatal life, mortality associated with IH in neonates was estimated with a CSMF of 2.06% (95% CI: 1.12–3.01).

The remaining ND-related deaths were broadly grouped as malignant and benign neoplasms of the brain and CNS, congenital malformations of the nervous system, other neurological disorders, and symptoms and signs involving the nervous and musculoskeletal systems. The mortality of these ND combined were estimated with a CSMF of 0.80% (95% CI: 0.56−1.04). Malignant and benign neoplasms of the brain and CNS were reported and estimated in individuals aged < 5 years with a CSMF of 0.23% (95% CI: 0.07–0.39). Congenital malformations of the nervous system were reported and estimated in children < 5 years with a CSMF of 0.52% (95% CI: 0.18–0.85).

## Discussion

This study provides the first detailed analysis of national data on neurological disorders in Tanzania. The study utilized a SAVVY approach developed to provide a standardized methodology in generating mortality estimates, causes of death and disease classification needed for local, regional and international comparability of mortality statistics. The adopted approach, which included national random samples of enumeration areas provided by the National Bureau of Statistics (NBS), multistage sampling methodology, use of two independent causes-of-death certifiers and ICD-10 guarantee credibility of the study's findings.

The main neurological disorders identified were cerebrovascular diseases, epilepsy, meningitis, cerebral palsy and other paralytic syndromes, and intrauterine hypoxia. Cerebrovascular disorders were the leading cause of death, most of which were attributed to stroke. We estimated that the number of neurological deaths in Tanzania ranged from 18,000 to 22,000 in 2012.

### Cerebrovascular diseases

Hypertensive diseases are the major risk factor for ND, specifically stroke and dementia [29]. The STEPwise approach to Surveillance (STEPS) survey designed by WHO estimated high blood pressure (>140/90 mmHg) in 25.9% of adults aged 25–64 years in Tanzania in 2012 [30]. High mortality estimates attributed to hypertension and cerebrovascular disorder observed in SAVVY are incoherent with the prevalence of hypertension reported in STEPS survey. A recent hospital mortality study for Tanzania mainland reported an increase of stroke-related mortality to 27% between 2006–2010 and 2011–2015. In the study period from 2006 to 2015, deaths attributable to stroke were 3.1% while cardiorespiratory and cardio-circulatory diseases accounted for 6.6% and 5.6%, respectively [31]. Comparable estimates of cerebrovascular mortality by residency in this study indicate lifestyle-related diseases equally affect rural and urban residents. These observations deviate from the common knowledge that urban residents are at higher risk for NCD in general compared to rural residents. Insufficient coverage of human resources and medicine supply for cardiovascular diseases may also explain why Tanzania lags behind in slowing down the increasing trends in the majority of NCD. In 2014, the Tanzania Service Provision Assessment Survey (TSPA) indicated a lack of guidelines for healthcare providers for NCD services [32]. The TSPA indicated that less than 10% of facilities have providers who have recently received training in providing services for cardiovascular and other chronic diseases [32]. Despite the fact that majority of Tanzanians access their health services through dispensaries and health facilities, the TSPA indicated the that availability of essential medicines for cardiovascular and other chronic diseases is lower in most dispensaries and health facilities than in hospitals.

### Epilepsy

The population estimate of 7.3 (95% CI: 6.9–7.6) deaths per 100,000 was comparable to estimates in the three large demographic surveillance sites: 5.4 (95% CI: 4–6.7) in Rufiji, 7.9 (95% CI: 6.1–9.7) in Ifakara Rural and 3.9 (95% CI: 1.3–6.4) deaths per 100,000 population in Ifakara Urban [33]. Epilepsy mortality rate and CSMF were comparable to estimates reported elsewhere in most developing countries. The majority of interventions targeting epilepsy are those aimed at eliminating the *Taenia solium* tapeworm, which is responsible for the development of taeniosis, a major cause of preventable epilepsy [34,35]. In Tanzania, the National Schistosomiasis Control Program (NSCP) routinely implemented school-based mass drug administration targeting both schistosomiasis and *Taenia solium* [36]. Several interventions have been devised to reduce parasite infections in Tanzania; however, most of these interventions have proved to yield modest efficacy, and therefore need to be re-evaluated [37].

Despite these efforts, mortality of epilepsy has remained stable over the years in Tanzania with reference to the previous national demographic surveillance system’s study conducted in 1992–1995, which estimated an epilepsy mortality rate of 15 and 5 deaths per 100,000 population in males and females, respectively [38].

Probable contributors to stagnation in reduction of epilepsy mortality include the rise of cerebrovascular diseases attributed to epilepsy such as stroke, the increase in emerging infectious diseases such as HIV, and an increase in traumatic brain injuries as a result of wide use of bikes/motor bikes without a helmet and vehicles without a seatbelt. These factors probably explain why in this study we have more mortality due to epilepsy in adults than in children, who normally have more epilepsy in general in SSA, especially those originating from febrile seizures.

### Meningitis

The mortality of meningitis was low, accounting for less than <0.8% of all deaths and 1.5% in children aged younger than 5 years. Analysis of WHO-reported cases of meningitis in Africa identified 11 regions (the ‘meningitis belt’) that account for 90% of all meningitis in SSA (Tanzania not included) [39]. High meningitis mortality in children may be explained by the fact that the meningitis pathogen that accounts for most cases of acute bacterial meningitis affects neonates and children. Observed meningitis deaths in adults may mostly constitute AIDS or TB-related deaths based on the fact that meningitis (cryptococcal) is a leading cause of death among HIV-infected individuals in SSA and in the studied population, where the latest HIV prevalence was estimated in adults aged 15–49. However, the unexpected low mortality of meningitis in adults may be explained by the fact that meningitis may be reported as immediate but not the underlying cause of death. The analysis in this study focused on underlying causes of death.

### Cerebral palsy and other paralytic syndromes

Cerebral palsy is a lifelong disability in children of non-progressive brain damage which most likely occurred during the antenatal, perinatal, or early postnatal period. Clinical presentations include challenges in coordination, stiff and/or weak muscles, tremors, and in some cases problem s in sense organs and reasoning. The burden of CP in Africa is estimated at 2–2.5 cases per 1000 live births and globally affects 0.2% of neonates [40,41]. Improving care at birth may have potential in reducing perinatal adverse events that are likely to result in the development of this disorder. There are scarce data on mortality in cerebral palsy in Africa, hence this study provides valuable statistics for future studies of the disorder.

Mortality from paralytic syndromes (including hemiplegia and paraplegia) was more common in individuals older than 50 years. These disorders may be sequelae of stroke, degenerative diseases, or infection of the nervous system; therefore, further investigation of its epidemiology is needed.

### Intrauterine hypoxia

In general, IH is the form of birth asphyxia that affects the brain as a result of oxygen deprivation of the fetus [42]. Birth asphyxia is a more general term, and we anticipate that most clinicians would classify IH as birth asphyxia. Despite the possibility for misclassification, physicians were able to capture IH deaths, which were the second leading cause of neurological death in children under 5 years. Poor maternal health condition, fetus development, and adverse perinatal events are the major culprits for the development of these disorders [42]. Improving antenatal and care at birth by scaling-up facility delivery and improving basic and comprehensive emergency obstetric and newborn care may significantly reduce pregnancy- or birth-related neurological morbidities such as cerebral palsy and mortality.

### Comparison with modeled estimates

Our estimates were lower or higher than those reported by GBD reports (Table 5) [43]. The estimated CSMFs of cerebrovascular diseases were comparable to estimates from the GBD for adults aged 15–49 years and close to estimates for all ages. SAVVY estimates of deaths attributable to epilepsy and road traffic injuries were higher in all age categories and overall than those reported in the GBD. On the other hand, the GBD reported higher estimates for deaths attributable to meningitis. The observed differences may be linked to methodological limitations in both approaches; however, SAVVY estimates are more likely to be closer to true population estimates. A substantial proportion of data used in generating the GBD for Tanzania are those derived from health and demographic surveillance systems, most of which are located in poor rural areas [44,45]. Recently, the 2017 GBD report in Tanzania compiled cerebrovascular diseasesdata dated from 2010 to 1993 due to the limited number of studies and poor coverage of civil and vital registration systems [46].

**Table 5.** Cause-specific mortality fraction (%) comparison of neurological disorders by study.

	Age 0–4 years		Age 15–49 years		All ages	
	SAVVY	GBD	SAVVY	GBD	SAVVY	GBD
Cerebrovascular	0.02 (0.00–0.09)	0.07 (0.04–0.09)	1.20 (0.65–1.92)	1.23 (0.96–1.76)	1.64 (1.30–1.99)	3.16 (2.66–3.63)
Epilepsy	0.17 (0–0.37)	0.11 (0.08–0.14)	1.94 (1.24–2.64)	0.55 (0.43–0.69)	0.94 (0.68–1.20)	0.25 (0.22–0.30)
Meningitis	1.43 (0.88–1.99)	3.53 (2.30–6.07)	0.53 (0.16–0.91)	1.53 (1.06–2.23)	0.80 (0.56–1.04)	2.10 (1.61–2.94)
Road injuries	0.28 (0.04–0.54)	0.54 (0.36–0.84)	5.83 (4.64–7.01)	2.41 (1.89–2.99)	2.43 (2.01–2.84)	1.48 (1.29–1.67)

Legend: Data sources SAVVY: Analysis of Sample vital registration with verbal autopsy GBD: Extracted from Global Burden of Disease report

### Limitations

Missing and misclassifying neurological disorders was the main possible limitation of this study attributed to lack of clinical training in neurology among physicians conducting death certification. A substantial proportion of deaths were coded as malaria in this study; however, some of the clinical manifestations of cerebral malaria such as fever, vomiting, and convulsions are also clinical manifestations of neurological disorders such as meningitis and epilepsy. Other possible misclassifications include those related to misclassifying cerebrovascular disorder as hypertensive disease, or vice versa. We did not observe traumatic brain injuries or intracranial deaths in this study despite the high mortality of road injuries estimated at 2.4% in all ages and 5.8% (95% CI: 4.7–7.1) in adults aged 15–49 years (results not reported but provided for contextual reasoning). Road injuries are one of the main causes of brain injuries; therefore, lack of information from VA or medical records may have resulted in missing some traumatic brain injury deaths in this study [9,10,47]. With regards to comparability of this study across countries, regions, and internationally, adoptions of newer versions of ICD-10 and the tabulation list of neurological disorders are likely to result in different mortality estimates elsewhere [48]. The recent version of ICD-10 recommends grouping some hypertensive disorders as cerebrovascular deaths; such changes are likely to result in estimates that are incomparable to what was observed in this study [49].

## Conclusions

The SAVVY survey provided estimates mortality burden of neurological disorders in Tanzania to the level of zones. Cerebrovascular diseases, epilepsy, meningitis, cerebral palsy and other paralytic syndromes, and intrauterine hypoxia are the leading causes of neurological mortality in Tanzania. The SAVVY sampling design strengthens the study in terms of representativeness for the nation and reliability of cause-of-death determination, and provides national baseline data on epidemiological information on neurological disorders needed for prevention and intervention programs. These estimates are rare in most SSA; therefore, we believe our archived data sets will contribute to advancing knowledge of neurological disorders. On the other hand, the burden associated with morbidities of neurological disorders might be far higher than that of mortality. In the case of stroke, mortality rates and deaths have substantially declined in most countries; however, the number of people living with stroke-related disabilities has been increasing. Reliable morbidity data can only be obtained when there is routine healthcare services data of good quality and community-based surveys. Integrating measures of morbidities such as DALYs in SAVVY design may provide a complete picture of disease, social and economic burden not only of neurological disorders but also of other prevalent diseases.

## Paper context

In the context of low- and middle-income countries, it has been established that the majority of neurological disorders are linked to infections and brain injuries. However, in the context of epidemiological transition, we hypothesize that there is high emerging incidence of age-related neurological disorders such as dementia, cerebrovascular and heart diseases. We utilize a SAVVY study; a national representative sample and explore mortality of neurological disorders in Tanzania by sex, residence, and special geographic areas (zone).
